# Morphological mechanism allowing a parasitic leech, *Ozobranchus jantseanus* (Rhynchobdellida: Ozobranchidae), to survive in ultra-low temperatures

**DOI:** 10.1242/bio.058524

**Published:** 2021-07-09

**Authors:** Shengli Gu, Jianjun Liu, Lei Xiong, Jinxiu Dong, Entao Sun, Haoran Hu, Mengli Yang, Liuwang Nie

**Affiliations:** 1 The Provincial Key Lab of the Conservation and Exploitation Research of Biological Resources in Anhui, Life Science College, Anhui Normal University, Wuhu, Anhui 241000, China; 2 Department of Parasitology, Wannan Medical College, Wuhu, Anhui 241002, China

**Keywords:** *Ozobranchus jantseanus*, Cryoprotective dehydration/Vitrification, Hemispherical state, Frozen section staining, Scanning electron microscopy

## Abstract

*Ozobranchus jantseanus* is the largest metazoan known to survive in liquid nitrogen without pretreatment to date; however, the mechanism underlying this tolerance remains unclear. In this study, the first analyses of histological and morphological changes in normal, frozen, and dehydrated states were performed. Adults survived after direct placement in liquid nitrogen for 96 h, with a survival rate of approximately 86.7%. The leech could withstand rapid desiccation and its survival rate after rehydration was 100% when its water loss was below about 84.8%. After freezing, desiccation, and ethanol dehydration, the leech immediately formed a hemispherical shape. Particularly during drying, an obvious transparent glass-like substance was observed on surface. Scanning electron microscopy revealed many pores on the surface of the posterior sucker, creating a sponge-like structure, which may help to rapidly expel water, and a hemispherical shape may protect the internal organs by contraction and folding reconstruction in the anterior–posterior direction. A substantial amount of mucopolysaccharides on the surface and acid cells and collagen fibers in the body, all of which contained substantial polysaccharides, may play a key protective role during freezing. Our results indicate that the resistance of leeches to ultra-low temperatures can be explained by cryoprotective dehydration/vitrification strategies.

This article has an associated First Person interview with the first author of the paper.

## Abstract (precis)

**Summary:** The freeze tolerance mechanism of Ozobranchus jantseanus, the largest metazoan animal requiring no pretreatment that can survive in ultra-low temperature, was first studied from the perspective of morphology.

## INTRODUCTION

Some organisms can survive in harsh environments, including drought and low-temperature conditions, by physiological and biochemical adaptations (Sakurai et al., 2008). Cold tolerance has been widely reported in animals, including terrestrial hibernating amphibians and reptiles, polar fishes, many species of insects, and numerous invertebrates inhabiting both terrestrial and aquatic environments (e.g. Aarset, 1982; Zachariassen, 1985; Devries and Cheng, 2005; Cheng and Detrich, 2007; Costanzo et al., 2008). Research to date has suggested that there are three main cold tolerance strategies: freeze tolerance, freeze avoidance, and cryoprotective dehydration/vitrification (Storey and Storey, 2013; Sørensen and Holmstrup, 2011). The former two mechanisms are more common and widely known strategies; in the latter mechanism, extreme whole-body dehydration coupled with high cryoprotectant levels essentially completely reduces freezable water in the organism (Denlinger and Lee, 2010; Storey and Storey, 2012, 2013). This strategy is used by numerous invertebrates in polar and non-polar regions and mirror those used by anhydrobiotic taxa with high desiccation tolerance (Wharton, 2003; Cornette and Kikawada, 2011; Sørensen and Holmstrup, 2011). Some adaptive mechanisms used by cold-tolerant species are common, and some taxa use more than one strategy or even all three of the above strategies (Storey and Storey, 2013).

*Ozobranchus jantseanus* (Oka, 1912) is a freshwater blood-sucking leech that lives in the folds of the neck and limbs of *Mauremys reevesii* and *M. japonica*; its known distribution is limited to East Asia, Japan, and China. Suzuki et al. (2014) first reported that *O.* *jantseanus* has a surprisingly high tolerance to freezing and thawing, with 100% survival after the hydration state in active adults directly injected in liquid nitrogen for 24 h; all individuals could survive for up to 32 months at −90°C. These results demonstrated that the leech can survive efficiently at a faster cooling rate for a long time than that of other cryobiotic organisms. It was possible that *O. jantseanus* was exposed to extremely low temperatures within a very short period of time, insufficient for the initiation of metabolic pathways required for cryoprotection, and therefore novel cryotolerance mechanisms were employed. However, the mechanism underlying tolerance to ultra-low temperatures in *O. jantseanus* is not clear.

To resolve this issue, in this study, the freezing and desiccation tolerance of *O. jantseanus* were tested under laboratory conditions. Additionally, the first morphological analysis of the species was performed by light and electron microscopy in its normal, frozen, and dehydrated states. Hematoxylin and Eosin (HE), Periodic Acid Schiff (PAS), and Sudan IV lipid staining were used to evaluate frozen sections. Our three objectives were to (1) assess the capacity to tolerate freezing and desiccation, (2) characterize the morphological changes in response to ultra-low temperatures, (3) explore the mechanism underlying ultra-low temperature tolerance in *O. jantseanus*.

## RESULTS

### Reassessment of the capacity of *O. jantseanus* to tolerate freezing

**Fig. 1.**
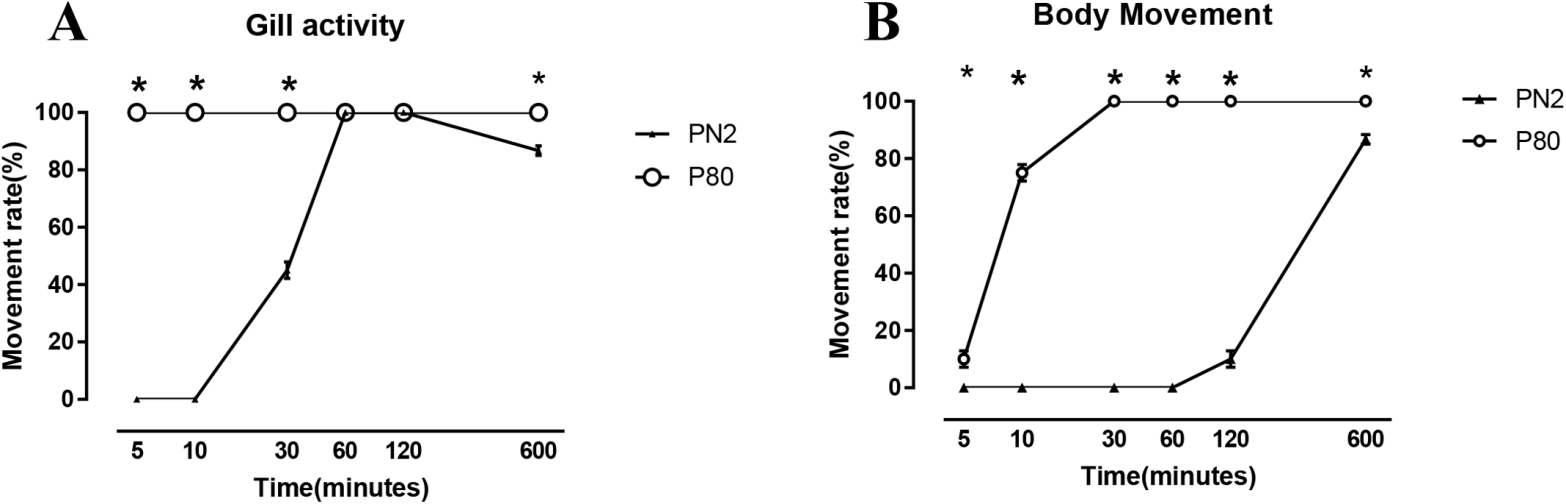
**Observation on gill activity and body movement of *Ozobranchus jantseanus* after freezing at −80°C and liquid nitrogen for 96 h. (A) χ^2^=120.00 (5 min), 120.00 (10 min), 45.52 (30 min); all *P*<0.01.** The gill activity data for resuscitated for 600 min after freezing were analyzed by Fisher's exact tests, *P*<0.01. (B) χ^2^=72.00 (10 min), 120.00 (30 min), 120.00 (60 min), 98.18 (120 min); all *P*<0.01. The data for body movement after resuscitation for 5 min and 600 min after freezing were analyzed by Fisher's exact test, both *P*<0.01. Values are presented as means±s.e.m. (*N*=20). Significant differences are indicated by an asterisk (*).

The results of an analysis of freezing tolerance are summarized in Fig. 1. In the P80 group, all individuals showed gill activity and body movement within 5 and 30 min, respectively, when returned to room temperature (about 20°C). In the PN2 group, after thawing and resuscitation in deionized water at room temperature, some individuals began to show gill activity after 10 min. The gill activity values for all individuals reached a maximum and some individuals began to show body movement after 60 min. However, after 600 min, 13.3±1.7% of the individuals lost gill activity and died.

### Water content and desiccation tolerance

**Fig. 2.**
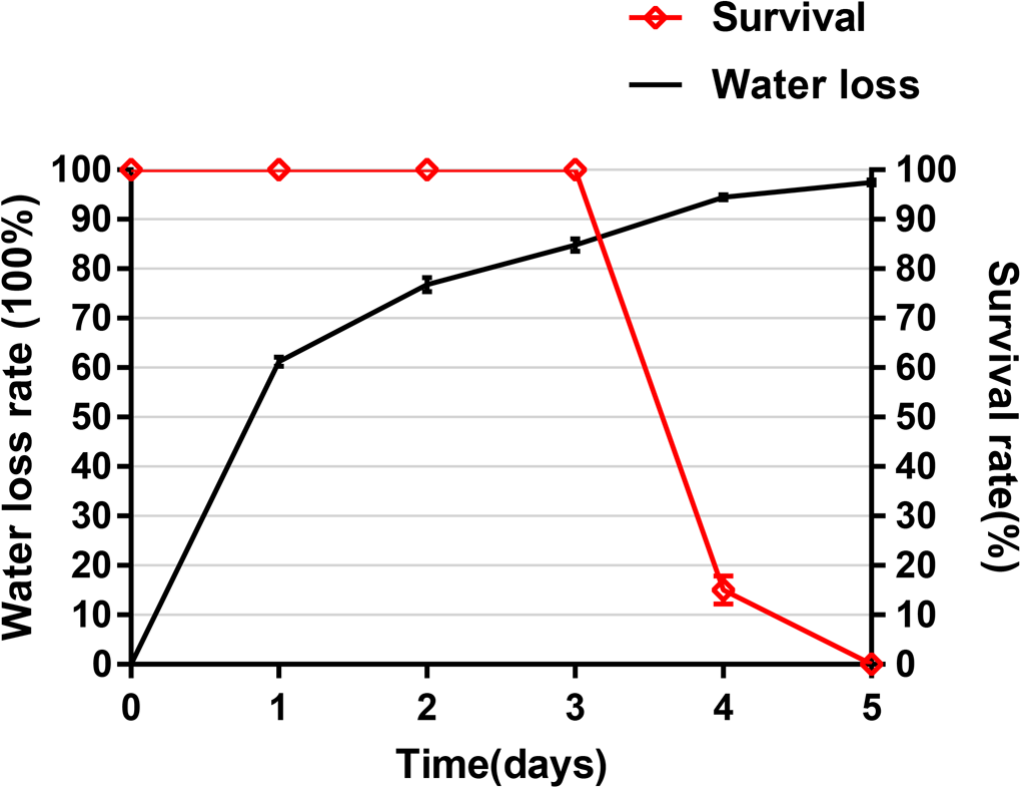
**Water loss and survival rates.***N*=20, Values are means±s.e.m. Two-tailed *t*-test and Pearson correlation analyses were used for data analyses. There was a significant negative correlation between the water loss and survival when the water loss was greater than 84.8±1.2% (*r*^2^=0.976, *P*<0.01).

The average water content of *O. jantseanus* was 74.9±2.9% in three experiments. Based on desiccation tolerance experiments, all individuals with water loss less than 84.8±1.2% were successfully resurrected. However, when the water loss increased to 94.5±0.5%, the survival rate dropped sharply to 15.0±2.9%, and when the water loss reached 97.4±0.5%, the gill activity stopped about 90 min after rehydration and all individuals died (see Fig. 2). During the experiment, the formation of a hemispherical shape was observed within 24 h after dehydration and the leech entered a completely static dormant state. Therefore, the effect of prolonged starvation on the survival rate can be ignored.

### Stereomicroscope observation of *O. jantseanus* in different environments

#### General morphology of *O. jantseanus*

**Fig. 3.**
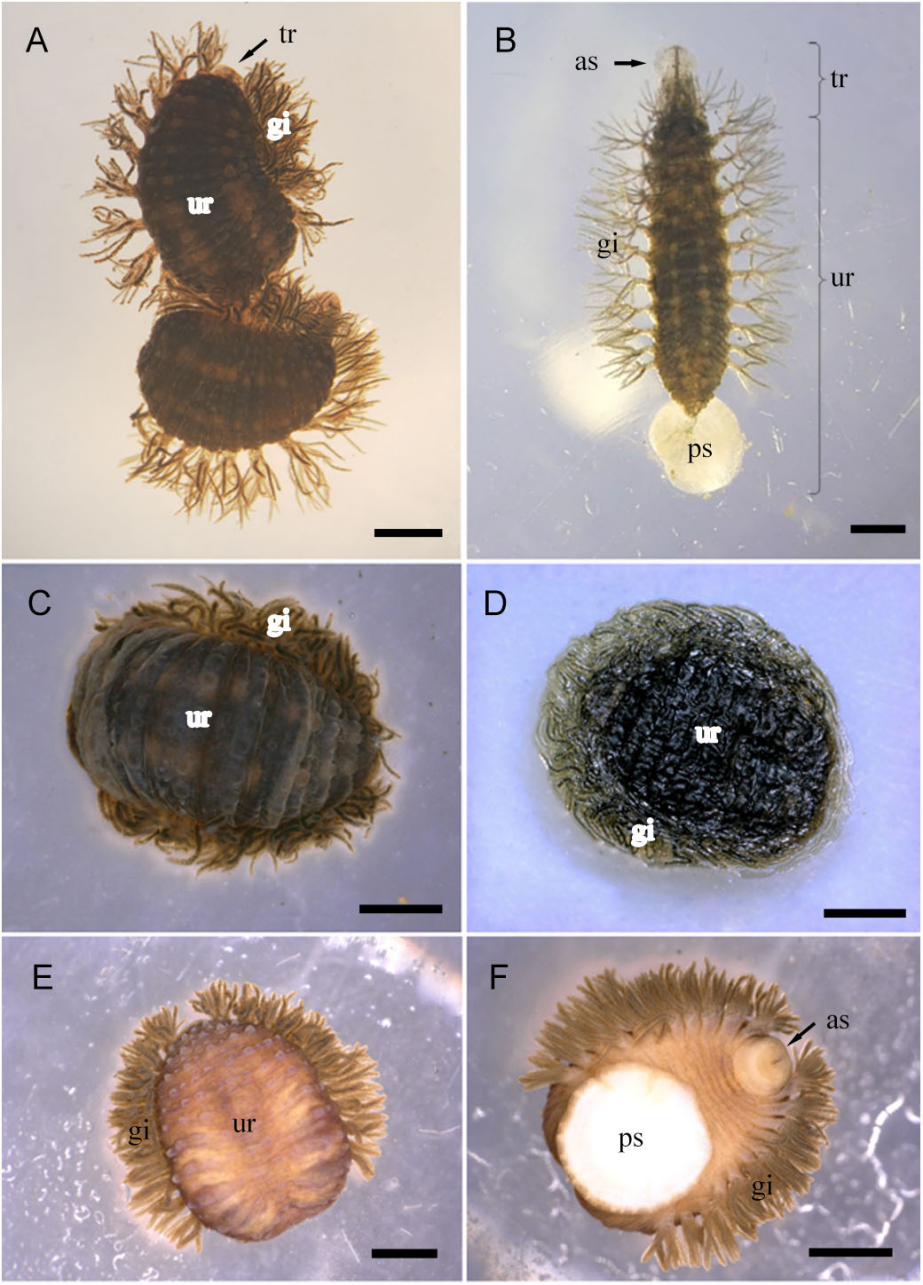
**Stereomicroscope observations of *O. jantseanus* in different conditions.** (A) Relaxed state. The trachelosome (tr) mostly retracts into the urosome (ur), only showing the eye part. (B) Motion state. Eleven pairs of branched gills (gi) are found on both sides of the leech and an anterior sucker (as) and posterior sucker (ps) are observed. (C) Frozen, hydrated state. (D) Dry, dehydrated state. A transparent glass-like substance could be seen on the surface of the leech body. (E) Dorsal view of the ethanol dehydration state. (F) Ventral view of the ethanol dehydration state. The contracted anterior sucker (as) and posterior sucker (ps) are visible. (C–F) Formation of the hemispherical state. Scale bars: A, 2000 µm; B–F, 1000 µm.

*O. jantseanus* is about 3–16 mm×1.2–7 mm in size. The brown body consists of a small subcylindrical neck (trachelosome) and a significantly broadened abdominal region (urosome) (Fig. 3B). The trachelosome often mostly retracts into the urosome, only showing the eye part (Fig. 3A). The anterior and posterior ends of the body have a small and large sucker, respectively. The posterior sucker diameter is equal to the dimeter of the body. The mouth is located on the front edge of the ventral side, in the center of the anterior sucker. There are 11 pairs of branched gills on both sides of the leech. The body segments of *O. jantseanus* consist of somites I–XXVII from the front end to the posterior sucker. Except for somites I to III and XXVII, each segment is divided into two annuli, a relatively large anterior annulus and slightly smaller posterior annulus.

#### Morphological observation of different environments of *O. jantseanus*

Under different environmental stresses (freezing, desiccation, and ethanol immersion), the morphological characteristics of *O. jantseanus* were similar (Fig. 3C–F). It completely retracted the trachelosome into the urosome, shrank, and folded all segments of the ventral surface of the urosome, exposing only part of the anterior annuli, and curled the posterior sucker to form a hemispherical state with a bulged back and flattened ventral surface (Fig. 3C–F). After 48 h of dehydration (at which point the water loss was 76.8±1.5%), in addition to the above-mentioned changes, a transparent glass-like substance was observed on the surface of the leech body (Fig. 3D).

**Fig. 4.**
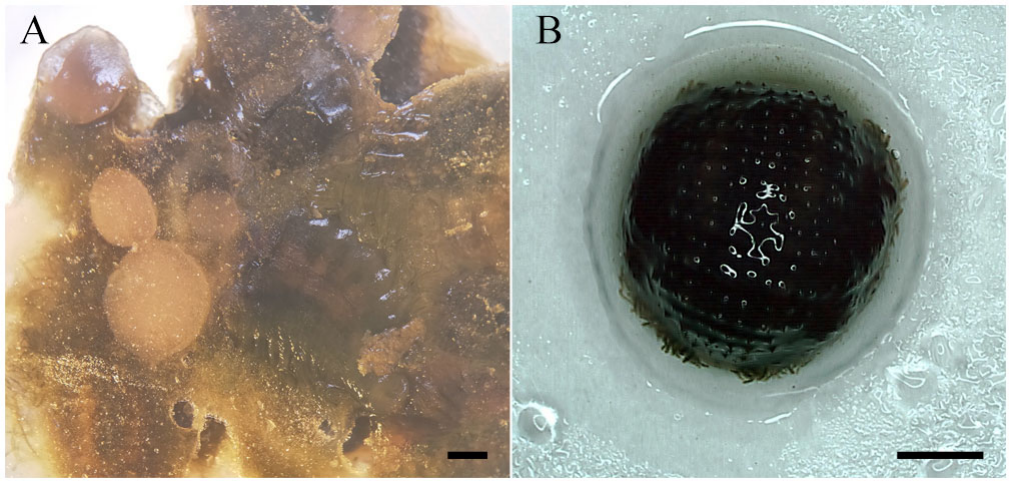
**A large amount of water was excreted from the body of *O. jantseanus* and formed ice.** (A) Many leeches. (B) Single leech. Water and a transparent glass-like substance formed around the hemispherical leech just after thawing. Scale bars: 1000 µm.

The surface moisture of *O. jantseanus* was absorbed by the filter paper before freezing at −80°C. After removal from the ultra-low temperature refrigerator, a substantial amount of water was discharged from the surrounding area to generate the freezing phenomenon (Fig. 4A,B).

#### SEM observation of *O. jantseanus*

**Fig. 5.**
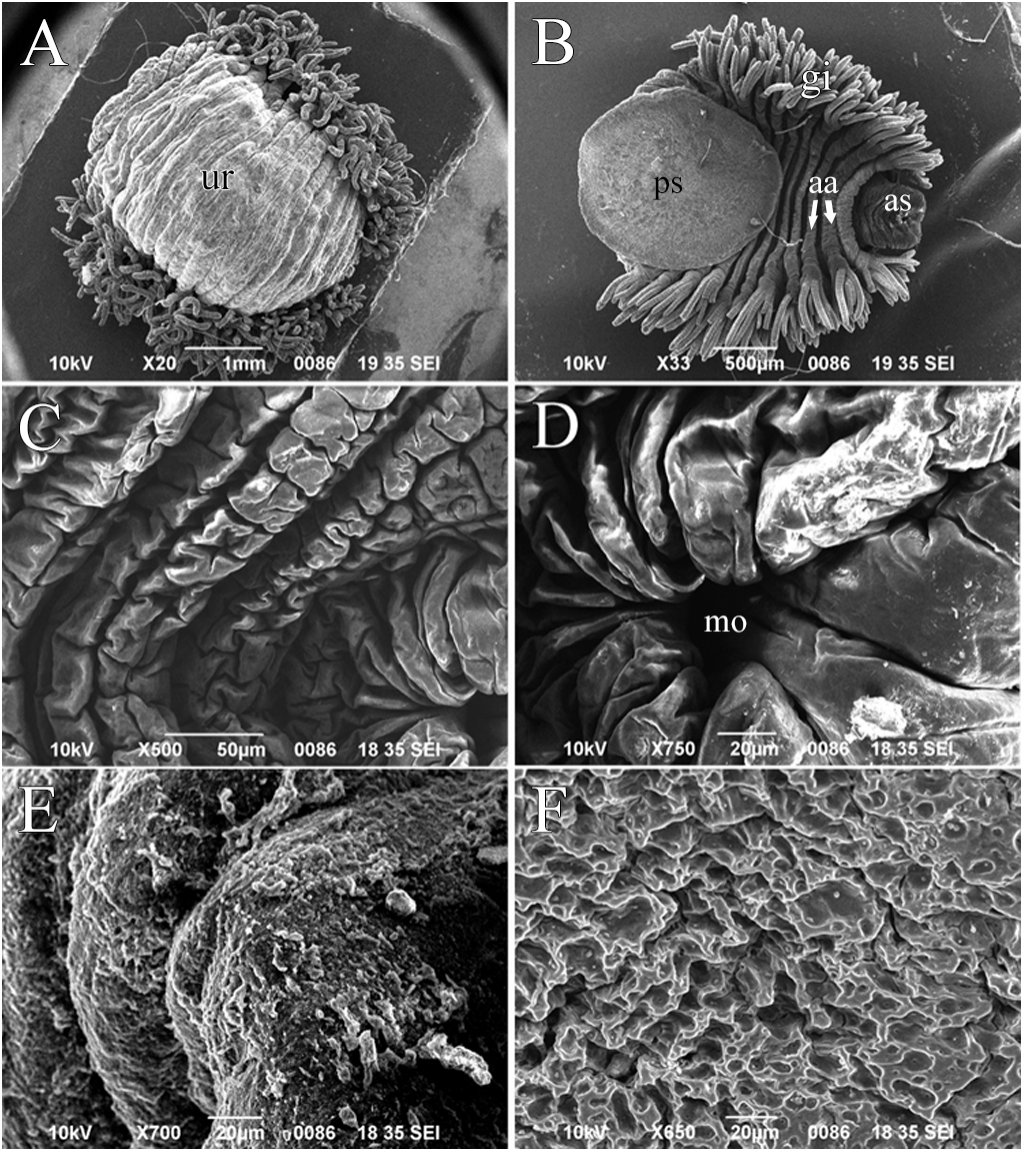
**Scanning electron micrographs of *O. jantseanus*.** (A) Entire body, dorsal view. Bulging on the back of the trachelosome (tr). (B) Entire body, ventral view. The posterior annulus was folded inside the anterior annulus (aa). (C) Trachelosome, ventral view. (D) Enlarged view of the mouth (mo). (E) Close-up of the dorsal view of *O. jantseanus*. (F) Close-up of the posterior sucker, ventral view. Scale bars: A, 1000 µm; B, 500 µm; C, 50 µm; D–F, 20 µm.

After dehydration and fixation with ethanol, *O. jantseanus* was in a hemisphere-like state and showed a raised back (Fig. 5A). Its ventral surface clearly contracted and the posterior annulus was folded inside the anterior annulus (Fig. 5B). The posterior sucker was curled to the ventral surface, and the whole ventral surface was almost completely flat (Fig. 5B). The annuli of the mouth also contracted and became concentrated and therefore the mouth sensillum could not be seen (Fig. 5C,D). There were a large number of mucopolysaccharide particles on the back surface of *O. jantseanus* (Fig. 5E) and many sponge-like holes on the surface of the posterior sucker with mucus particles attached (Fig. 5F).

### Frozen section observation of *O. jantseanus*

#### HE and PAS staining observation of frozen sections

**Fig. 6.**
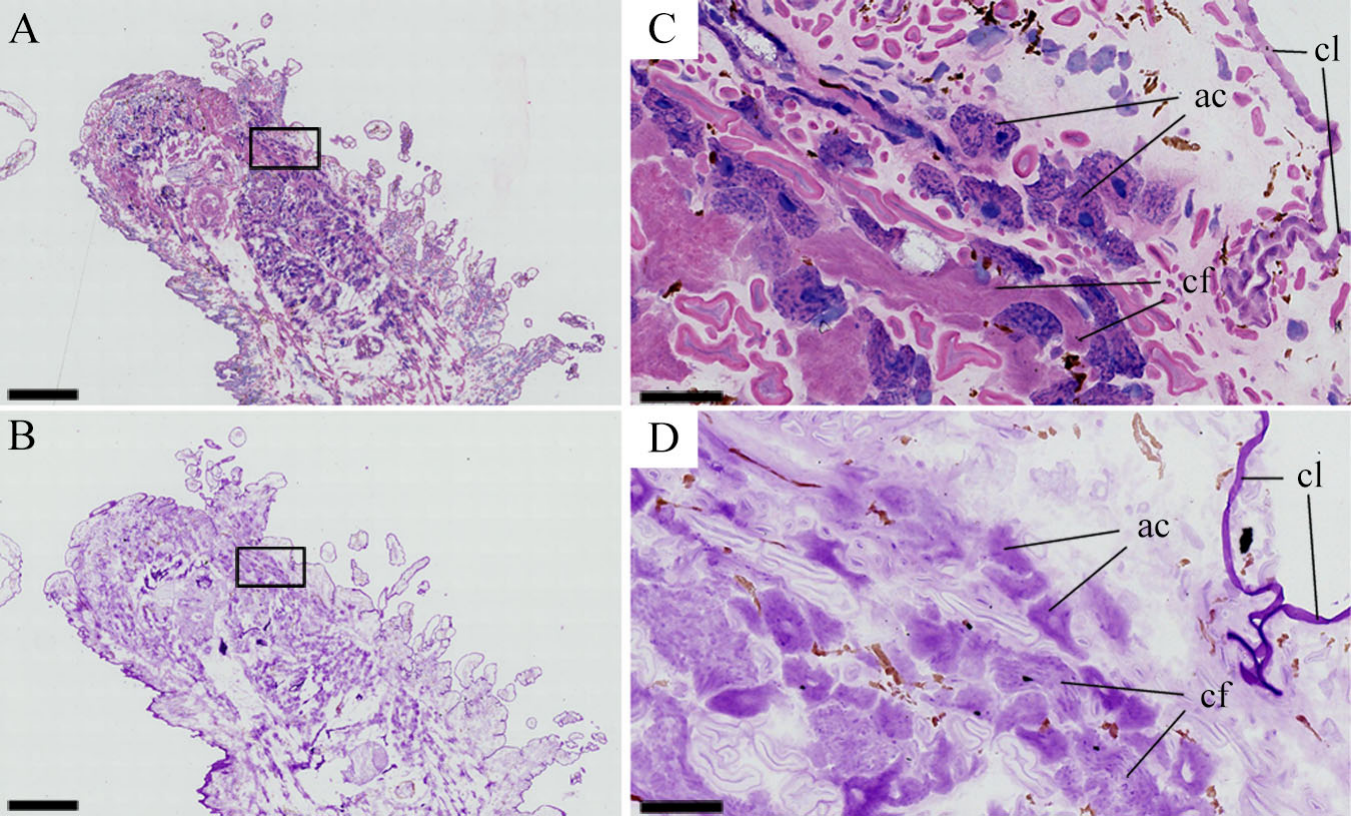
**HE and PAS staining of frozen sections of *O. jantseanus* on the coronal plane.** (A) HE staining. (B) PAS staining. (C) Close-up view of HE staining in the black frame of the image A. Dark blue acid cells (ac), red-stained collagen fibers (cf), and cuticle layer (cl). (D) Close-up view of PAS staining in the black frame of the image B. The darkest PAS staining was observed in the cuticle layer (cl), followed by acid cells (ac), and collagen fiber (cf). Scale bars: A and B, 500 µm; C and D, 50 µm.

Staining results for HE and PAS are shown in Fig. 6. The darkest PAS staining corresponded to the mucopolysaccharides outside of the cuticle layer, followed by a large number of acid cells and collagen fibers distributed in the tissue space (Fig. 6D). Basophilic granules were stained by HE in acid cells, while the nuclei of acid cells did not show positive polysaccharide staining.

#### Lipid staining observation using frozen sections

**Fig. 7.**
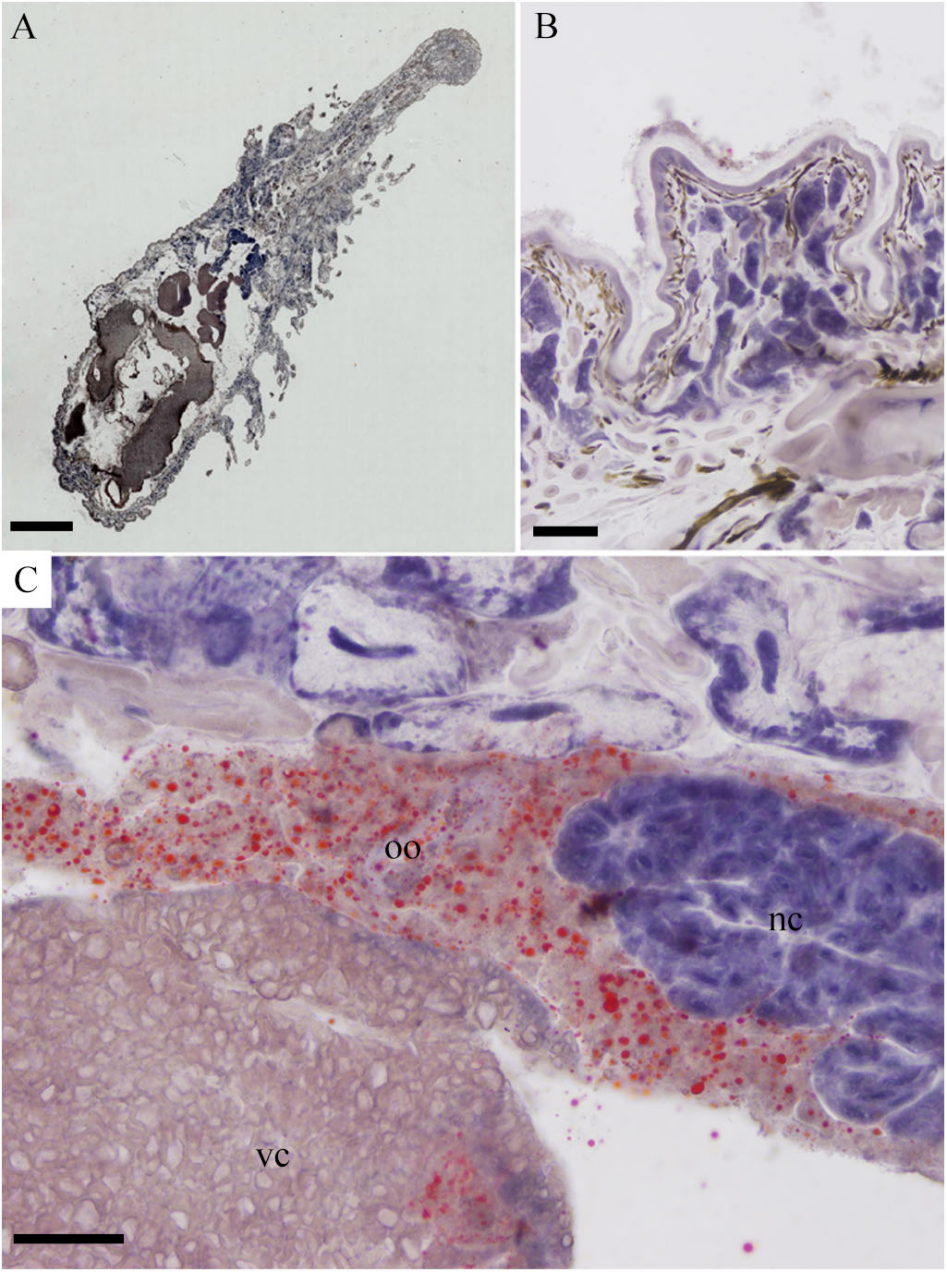
**Sudan IV staining of frozen sections of *O. jantseanus* on the coronal plane.** (A) General view of *O. jantseanus*. Substantial accumulation of lipid droplets was not observed. (B) Close-up view of the epidermal tissue. No lipid droplets were found. (C) Close-up view of the splanchnic tissue. Orange lipid droplets were found in the immature oocytes (oo), and a few lipid droplets were detected in vitelline cells (vc). No lipid droplets were detected in nurse cells (nc). Scale bars: A, 1000 µm; B-D, 50 µm.

The lipid staining results for frozen section are shown in Fig. 7. Substantial accumulation of lipid droplets in the whole body was not observed (Fig. 7A) and lipid droplets were not detected by local epidermal tissue staining (Fig. 7B). Only orange lipid droplets were found in immature oocytes of the ovary, lipid droplets were occasionally found in vitelline cells, and no lipid droplets were found in nurse cells (Fig. 7C).

## DISCUSSION

### Capacity of *O. jantseanus* to tolerate freezing and desiccation

In this study, the long-term freezing of liquid nitrogen (96 h) verified the ultra-low temperature tolerance of *O. jantseanus*, and the observation of spawning after resuscitation further indicated that freezing has no effect on the long-term survival and reproductive ability. In short, in the early stage of the recovery period, the −80°C group recovered more quickly than the liquid nitrogen group, but there was no significant difference in long-term survival between the two groups after resuscitation. Suzuki et al. (2014) reported that *O. jantseanus* shows 100% survival in active adults after hydration following treatment in liquid nitrogen for 24 h, however, long-term survival after freezing was not reported. Compared with other species able to tolerate liquid nitrogen freezing, such as *Drosophila* larvae, the African chironomid, tardigrades, rotifers, and some nematodes (e.g. Hinton, 1960; Kelly and Campbell, 1974; King et al., 1983; Koštál et al., 2011; Wełnicz et al., 2011), *O. jantseanus* evaluated in this study had the longest freezing time in liquid nitrogen (96 h) reported to date. Most species able to tolerate liquid nitrogen freezing need several days of dehydration and anhydrobiosis to survive in such conditions with a high resuscitation rate (e.g. Hinton, 1960; Ricci et al., 2003; Horikawa et al., 2008; Koštál et al., 2011). However, Ramløv and Westh (1992) found that the survival rate of the hydrated tardigrade *Adorybiotus coronifer* is about 55% after resuscitation under low cooling rates, with 100% lethality under a cooling rate of 1500°C min^−1^. Different from these analyses of tardigrades, the leech was placed directly in liquid nitrogen for 96 h, with an estimated cooling rate of 1500°C min^−1^ (Ramløv and Westh, 1992) and a survival rate after recovery of about 86.7%, further indicating that the hydrated leech was more tolerant to ultra-low temperatures under super-fast cooling. In addition, it is the largest ultra-low temperature-resistant metazoan reported to date.

According to Yang (1996), *O. jantseanus* can lose 4/5 of its body water and recover after being placed in water for several days, indicating that it is also a dehydration-tolerant species. However, Suzuki et al. (2014) reported that *O. jantseanus* cannot survive under dry conditions. In this study, the relationship between the water loss and survival rate of *O. jantseanus* was reported for the first time. Our experimental data proved that *O. jantseanus* has strong dehydration tolerance. In addition, Nakahara et al. (2008) found that the rehydration of *P. vanderplanki* under high humidity and slow dehydration conditions (100% RH for 48 h followed by 5% RH for 48 h) has the highest survival rate, and after 48 h of dehydration, about 80% of larvae subjected to the desiccation initially survived, but less than 30% of larvae were alive 48 h later. No larvae were recovered when desiccated at 93% or 85% RH for 48 h followed by 5% RH. Therefore, the authors speculated that slow dehydration within the first 48 h may be required for the synthesis and distribution of essential molecules for anhydrobiosis. However, the survival rate of *O. jantseanus* in the first 2 days (85% RH for 48 h) was 100%. Moreover, the survival rates of rehydrated tardigrades *Ramazzottius oberhaeuseri* and *Echiniscus* spp. after drying at room temperature at the beginning of the experiment were 91.1% and 71.7%, respectively (Bertolani et al., 2004). To some extent, *O. jantseanus* can tolerate rapid drying for a short duration.

*P. vanderplanki* larvae and some tardigrades do not live at high latitudes. Some are rarely exposed to ultra-low temperatures and accordingly the observed tolerance arose by non-adaptive evolution; that is, ultra-low temperature tolerance is a by-product of desiccation tolerance. For example, the larvae of *P. vanderplanki* (Hinton, 1960) live in small caves or puddles and often experience repeated dehydration and hydration in nature, resulting in the evolution of the ability to withstand desiccation, and only dehydrated *P. vanderplanki* larvae can withstand ultra-low temperatures (Hinton, 1960). *O. jantseanus* is a permanent parasite of *Mauremys reevesii*, which inhabits swamps, ponds, streams, wetlands. The turtles often sunbathe on rocks or fallen trees near the water (Zhao and Adler, 1993; Yin et al., 2016). *O. jantseanus* may be subjected to frequent dehydration and rehydration resulting from the movement of the host *M. reevesii* into and out of the water, thus resulting in the evolution of the ability to respond quickly to dehydration. Its low temperature tolerance may also be associated with this desiccation tolerance.

### Morphological response of *O. jantseanus* in ultra-low temperatures

*O. jantseanus* have similar morphological characteristics to those of the ‘tun’ state of Tardigrada (Horikawa et al., 2008; Halberg et al., 2013) and develop a hemispherical state characterized by a dorsal bulge and full ventral contraction and flattening under ultra-low temperatures, desiccation, and ethanol.

*O. jantseanus* is the only reported leech that can tolerate ultra-low temperatures, which is unlike other taxa in the subclass Hirudinea. *Ozobranchus margoi* and *O. jantseanus,* belonging to the same genus, have similar morphologies; however, *O. margoi* is not tolerant to freezing (Suzuki et al., 2014). In addition, it has only a C-shaped curl at both ends and does not show a hemispherical form after dehydration by ethanol (Cascarano et al., 2017); however, *O. jantseanus* can form a hemispherical shape, with similar physical changes to those of many anhydrobiotic animals, such as tardigrades and rotifers (Wright, 2001; Ricci et al., 2003). Indeed, these changes can reduce the evaporation of water to protect internal organs and cells (Wright, 1989a,b; Halberg et al., 2013) because when tardigrades enter anhydrobiosis, low temperature tolerance increases substantially (Horikawa et al., 2008). These morphological characteristics of *O. jantseanus*, which were similar to those of anhydrobiotic organisms, may also be a protective mechanism against environmental pressure. Similarly, *O. jantseanus* may quickly remove water from its body by contraction at ultra-low temperatures to reduce damage caused by ice crystals. Moreover, this rapid reconstruction of morphology (hemispherical state) is beneficial to protect internal organs from ultra-low temperatures.

### Mechanism underlying cryoprotective dehydration/vitrification

We observed a large number of acid cells (see Fig. 6C,D) with high polysaccharide contents in the tissue space of *O. jantseanus*. The polysaccharide content was highest on the body surface and in acid cells, both of which are acidic, and collagen fibers also contained polysaccharides. Notably, the polysaccharide composition of acid cells may be the same as the acid mucopolysaccharides on the body surface (Yang, 1996). In this experiment, a large number of acidic mucopolysaccharides were found. Mucopolysaccharides are a combination of polysaccharides and protein, which is a long unbranched polysaccharide composed of a repetitive disaccharide unit (Varki et al., 2009). This acidic mucopolysaccharide component and other unknown polysaccharides may protect cells, fill tissue gaps, and these glass-like substances observed in freezing and desiccation experiment may act as vitrification matrix components along with bound proteins under low temperatures and in dry conditions. Accordingly, they play important roles in resistance to low temperatures and desiccation.

Previous studies have shown that ultra-low temperatures in some organisms are closely related to vitrification, which is related to the accumulation of trehalose (Crowe et al., 1998; Storey and Storey, 2013). The larvae of *P. vanderplanki* accumulate a large amount of trehalose during drying, accounting for up to 20% of their body weight, and this replaces water in the tissues to protect cells (Clegg, 1965; Watanabe et al., 2002; Sakurai et al., 2008; Cornette and Kikawada, 2011). Trehalose in the dehydrated nematode *Aphelenchus avenae* also reaches 15% of the dry weight (Crowe and Madin, 1975). However, some organisms contain very little trehalose (Hengherr et al., 2008). Some tardigrades use protein as a vitrification matrix, and non-crystalline amorphous solids (vitrification) are formed by tardigrade-specific intrinsically disordered proteins after drying, which confer a protective effect (Yamaguchi et al., 2012; Boothby et al., 2017; Fukuda et al., 2017). In addition, some protective proteins have been identified in *P. vanderplanki* (Hatanaka et al., 2013; Furuki et al., 2007). The glass-like substance, which probably plays a protective role in drying and freezing, found in this experiment may be composed of mucopolysaccharides. Further studies are needed to determine the specific composition.

In fact, *O. jantseanus* is the only animal with 100% survival after direct placement in liquid nitrogen for 24 h (Suzuki et al., 2014) discovered to date. The results of this experiment further proved that the species has a high recovery rate (86.7±1.7%) after long-term exposure to liquid nitrogen. Furthermore, *O. jantseanus* is the largest known ultra-low temperature tolerant animal. The ability of the leech to tolerate rapid ultra-low temperatures is closely related to its tolerance to rapid drying. Extensive research has focused on tardigrades and the African chironomid, they were even taken to outer space as model organisms in aerospace research (Jönsson et al., 2008; Novikova et al., 2011). However, little is known about the morphology and anti-freezing mechanism of *O. jantseanus*. The species is a very good model for studies of ultra-low temperature tolerance and provides additional data for resolving related scientific issues, such as the resuscitation of entire living organisms after freezing. However, further research is needed to determine the specific mechanism underlying rapid dehydration and the specific composition of the glass-like substance is still unclear.

## MATERIALS AND METHODS

### Sample collection and preparation

*O. jantseanus* individuals were collected from their hosts, the freshwater turtle *M. reevesii*, at a turtle farm in Wuhu City (118°45′ 31°48′), China. Adult leeches (length >3 mm) in the neck and limb folds of turtles were manually removed with forceps and the turtles were returned to the pool immediately after sampling. The experiment did not cause any harm to turtles. The leeches were washed four times in ddH_2_O at room temperature (about 20°C) and wiped superficial water on the body just before each experiment.

### Reassessment of freezing tolerance in *O. jantseanus*

A total of 40 adult leeches were randomly divided into two groups, i.e., a liquid nitrogen group (PN2) and −80°C group (P80), with 20 individuals per group. This experiment was repeated three times. The samples were put in a 2 ml cell cryopreservation tube (Eppendorf, Premium U570-86) and directly placed in a −80°C ultra-low temperature refrigerator or liquid nitrogen immersion. After 96 h, the leeches were removed and placed in deionized water at room temperature to thaw and resuscitate. Gill activity and body movement were observed under a stereomicroscope after 5, 10, 30, 60, 120, and 600 min, and the two parameters were recorded separately.

### Desiccation tolerance in *O. jantseanus*

A total of 20 individuals were placed in a 1.5 ml EP tube with pinholes to calculate the water content of the leech. As described by Nakahara et al. (2008), the pre-weighed leeches were completely desiccated in an oven (100°C) for 24 h and then re-weighed using an electronic balance. The water content was defined as follows: water content (%)=(Wet weight - Dry weight)/Wet weight×100. The experiment was repeated three times.

A total of 20 Petri dishes with 20 individuals each were weighed separately to calculate the wet weight before testing. A two-step drying method was performed, following the methods described by Sakurai et al. (2008). All Petri dishes were placed a vacuum glass desiccator with a diameter of 300 mm. Humidity in the desiccator was controlled with 500 ml of saturated salt solution KCl and 400 g of silica gel. In the first step, the leeches were dehydrated with KCl-saturated saline for 48 h. In the second step, silica gel desiccant was used to further drying for 72 h. The relative humidity was measured using a thermo-hygrometer. The relative humidity was 85% for 48 h and then 25% for 72 h. During this process, three Petri dishes were taken every day and weighed. The water loss was calculated as followed: Water loss (%)=(wet weight - weight after drying on the day)÷(wet weight×water content)×100. Then, the dehydrated leeches were hydrated in deionized water, and the number of surviving leeches after hydration for 24 h was recorded.

### Stereomicroscopy observation of the leech in different environments

The leeches were divided into four groups (*n*=10). Leeches in the untreated group were placed directly into deionized water. Leeches in the frozen group were frozen at −80°C for 2 h and then placed in deionized water. Leeches in the desiccation group were dehydrated at 85% humidity for 48 h. Leeches in the ethanol-treated group leeches were immersed in ethanol for gradient dehydration (50%, 70%, 80%, 90%, ascending ethanol, 15 min per step, followed by 100% ethanol for 30 min). Body shapes were immediately observed under a stereomicroscope after processing.

### Scanning electron microscopy (SEM) observation

Ten individuals were processed according to the methods described by Cascarano et al. (2017). Briefly, leeches were fixed in 10 ml of 2.5% glutaraldehyde (Solarbio, San Diego, CA, USA) for 3 h. Then, the leeches were dehydrated in 50%, 70%, 80%, 90%, and 100% ethanol, for 15 min per step, followed by preservation in 100% ethanol. Finally, the pre-treated leeches were mounted on an electron microscope copper table with conductive glue in the bracket after spraying with gold-palladium and observed by SEM (JEOL JSM-6390LV).

### Observation of *O. jantseanus* frozen sections

The posterior sucker of the leech was fixed with frozen section glue and then the body was quickly stretched using tweezers to prevent the development of a hemispherical form. The body was immediately fixed with the glue completely and sliced along the coronal plane at a thickness of 5 μm per each layer using a frozen microtome (CM1950; LEICA, Wetzlar, Germany). The frozen sections were stained with HE (refer to Scouten, 2010), PAS (operated according to the standard protocol of Periodic Acid Schiff/PAS Stain Kit, Baso, Shenzhen, PRC), and Sudan IV (refer to Lloyd et al., 2008) and observed and analyzed using an automatic digital slice scanning microscope (Motic BA600Mot).

### Statistical analyses

GraphPad Prism 7.0 (GraphPad Software, Inc., La Jolla, CA, USA) was used to generate plots and to calculate means and standard error of mean (s.e.m.) for statistical tests. All values are presented as means±s.e.m. from three experiments. All statistical analyses were performed using SPSS 18.0 (IBM, Armonk, NY, USA). The Chi-squared test was used for the analysis of gill activity and body movement after resuscitation from freezing. Two-tailed *t-*tests followed by Pearson correlation analyses were used for the analysis of desiccation tolerance. *P-*values of <0.05 were statistically significant.

## References

[BIO058524C1] Aarset, A. V. (1982). Freezing tolerance in intertidal invertebrates (a review). *Comp. Biochem. Phys. A.* 73, 571-580. 10.1016/0300-9629(82)90264-X

[BIO058524C2] Bertolani, R., Guidetti, R., Jönsson, K. I., Altiero, T., Boschini, D. and Rebecchi, L. (2004). Experiences with dormancy in tardigrades. *J. Limnol.* 63, 16-25. 10.4081/jlimnol.2004.s1.16

[BIO058524C3] Boothby, T. C., Tapia, H., Brozena, A. H., Piszkiewicz, S., Smith, A. E., Giovannini, I., Rebecchi, L., Pielak, G. J., Koshland, D. and Goldstein, B. (2017). Tardigrades use intrinsically disordered proteins to survive desiccation. *Mol. Cell.* 65, 975-984.e5. 10.1016/j.molcel.2017.02.018 28306513 PMC5987194

[BIO058524C4] Cascarano, M. C., Keklikoglou, K., Arvanitidis, C. and Katharios, P. (2017). Contribution to the morphological description of the marine leech, *Ozobranchus margoi* (Apáthy) (Rhynchobdellida: Ozobranchidae) by using combined histology, micro-CT and SEM. *Zootaxa* 4337, 91-108. 10.11646/zootaxa.4337.1.4 29242432

[BIO058524C5] Cheng, C.-H. C. and Detrich, H. W. (2007). Molecular ecophysiology of antarctic notothenioid fishes. *Philos. Trans. R. Soc. B Biol. Sci.* 362, 2215-2232. 10.1098/rstb.2006.1946 PMC2443173 17553777

[BIO058524C6] Clegg, J. S. (1965). The origin of threhalose and its significance during the formation of encysted dormant embryos of *Artmia salina*. *Comp. Biochem. Physiol.* 14, 135-143. 10.1016/0010-406X(65)90014-9 14288194

[BIO058524C7] Cornette, R. and Kikawada, T. (2011). The induction of anhydrobiosis in the sleeping chironomid: current status of our knowledge. *IUBMB Life.* 63, 419-429. 10.1002/iub.463 21547992

[BIO058524C8] Costanzo, J. P., Lee, R. E. and Ultsch, G. R. (2008). Physiological ecology of overwintering in hatchling turtles. *J. Exp. Zool. A. Ecol. Integr. Physiol.* 309A, 297-379. 10.1002/jez.460 18484621

[BIO058524C9] Crowe, J. H. and Madin, K. A. C. (1975). Anhydrobiosis in nematodes: evaporative water loss and survival. *J. Exp. Zool.* 193, 323-3334. 10.1002/jez.1401930308

[BIO058524C10] Crowe, J. H., Carpenter, J. F. and Crowe, L. M. (1998). The role of vitrification in anhydrobiosis. *Annu. Rev. Physiol.* 60, 73-103. 10.1146/annurev.physiol.60.1.73 9558455

[BIO058524C11] Denlinger, D. L. and Lee, R. E. (2010). *Low Temperature Biology of Insects*. Cambridge University Press. 10.1017/CBO9780511675997

[BIO058524C12] Devries, A. L. and Cheng, C.-H. C. (2005). Antifreeze proteins and organismal freezing avoidance in polar fishes. *Fish Physiol.* 22, 155-201. 10.1016/S1546-5098(04)22004-0

[BIO058524C13] Fukuda, Y., Miura, Y., Mizohata, E. and Inoue, T. (2017). Structural insights into a secretory abundant heat-soluble protein from an anhydrobiotic tardigrade, *Ramazzottius varieornatus*. *FEBS Lett.* 591, 2458. 10.1002/1873-3468.12752 28703282

[BIO058524C14] Furuki, T., Miyazawa, M., Kikawada, T., Okuda, T. and Sakurai, M. (2007). 3p020 effects of drying history on the conformational states of a dehydrated LEA protein from *Polypedilum vanderplanki*(proteins-structure and structure-function relationship, poster presentations. *Seibutsu Butsuri* 47, S208. 10.2142/biophys.47.S208_1

[BIO058524C15] Halberg, K. A., Jørgensen, A. and Møbjerg, N. (2013). Desiccation tolerance in the tardigrade *Richtersius coronifer* relies on muscle mediated structural reorganization. *PLoS ONE* 8, e85091. 10.1371/journal.pone.0085091 24391987 PMC3877342

[BIO058524C16] Hatanaka, R., Hagiwara-Komoda, Y., Furuki, T., Kanamori, Y., Fujita, M., Cornette, R., Sakurai, M., Okuda, T. and Kikawada, T. (2013). An abundant LEA protein in the anhydrobiotic midge, PvLEA4, acts as a molecular shield by limiting growth of aggregating protein particles. *Insect Biochem. Mol.* 43, 1055-1067. 10.1016/j.ibmb.2013.08.004 23978448

[BIO058524C17] Hengherr, S., Heyer, A. G., Köhler, H. R. and Schill, R. O. (2008). Trehalose and anhydrobiosis in tardigrades - evidence for divergence in responses to dehydration. *FEBS J.* 275, 281-288. 10.1111/j.1742-4658.2007.06198.x 18070104

[BIO058524C18] Hinton, H. E. (1960). A Fly larva that tolerates dehydration and temperatures of −270° to +102°C. *Nature* 188, 336-337. 10.1038/188336a0

[BIO058524C19] Horikawa, D. D., Kunieda, T., Abe, W., Watanabe, M., Nakahara, Y., Yukuhiro, F., Sakashita, T., Hamada, N., Wada, S., Funayama, T. et al. (2008). Establishment of a rearing system of the extremotolerant tardigrade *Ramazzottius varieornatus*: a new model animal for astrobiology. *Astrobiology* 8, 549-556. 10.1089/ast.2007.0139 18554084

[BIO058524C21] Jönsson, K. I., Rabbow, E., Schill, R. O., Harms-Ringdahl, M. and Rettberg, P. (2008). Tardigrades survive exposure to space in low Earth orbit. *Curr. Biol.* 18, R729-R731. 10.1016/j.cub.2008.06.048 18786368

[BIO058524C22] Kelly, J. D. and Campbell, W. C. (1974). Survival of *Nippostrongylus brasiliensis* larvae after freezing over liquid nitrogen. *Int. J. Parasitol.* 4, 173-176. 10.1016/0020-7519(74)90101-5 4856648

[BIO058524C23] King, C. E., Bayne, H. B., Cannon, T. K. and King, A. E. (1983). Cryopreservation of monogonont rotifers. *Hydrobiologia* 104, 85-88. 10.1007/BF00045956

[BIO058524C24] Koštál, V., Zahradníčková, H. and Šimek, P. (2011). Hyperprolinemic larvae of the drosophilid fly, *Chymomyza costata*, survive cryopreservation in liquid nitrogen. *Proc. Natl. Acad. Sci. USA* 108, 13041-13046. 10.1073/pnas.1107060108 21788482 PMC3156168

[BIO058524C25] Lloyd, D. J., McCormick, J., Helmering, J., Kim, K. W., Wang, M., Fordstrom, P., Kaufman, S. A., Lindberg, R. A. and Véniant, M. M. (2008). Generation and characterization of two novel mouse models exhibiting the phenotypes of the metabolic syndrome: apob48^−/−^lep^ob/ob^ mice devoid of apo^E^ or Ldlr. *Am. J. Physiol. Endocrinol. Metab.* 294, E496-E505. 10.1152/ajpendo.00509.2007 18160459

[BIO058524C26] Nakahara, Y., Watanabe, M., Fujita, A., Kanamori, Y., Tanaka, D., Iwata, K. I., Furuki, T., Sakurai, M., Kikawada, T. and Okuda, T. (2008). Effects of dehydration rate on physiological responses and survival after rehydration in larvae of the anhydrobiotic chironomid. *J. Insect Physiol.* 54, 1220-1225. 10.1016/j.jinsphys.2008.05.007 18652833

[BIO058524C27] Novikova, N., Gusev, N., Polikarpov, N., Deshevaya, E., Levinskikh, M., Alekseev, V., Okuda, T., Sugimoto, M., Sychev, V. and Grigoriev, А. (2011). Survival of dormant organisms after long-term exposure to the space environment. *Acta Astronaut* 68, 1574-1580. 10.1016/j.actaastro.2010.05.019

[BIO058524C28] Ramløv, H. and Westh, P. (1992). Survival of the cryptobiotic eutardigrade *Adorybiotus coronifer* during cooling to −196°C: effect of cooling rate, trehalose level, and short-term acclimation. *Cryobiology* 29, 125-130. 10.1016/0011-2240(92)90012-Q

[BIO058524C29] Ricci, C., Melone, G., Santo, N. and Caprioli, M. (2003). Morphological response of a bdelloid rotifer to desiccation. *J. Morphol.* 257, 246-253. 10.1002/jmor.10120 12833383

[BIO058524C30] Sakurai, M., Furuki, T., Akao, K.-I., Tanaka, D., Nakahara, Y., Kikawada, T., Watanabe, M. and Okuda, T. (2008). Vitrification is essential for anhydrobiosis in an African chironomid, *Polypedilum vanderplanki*. *Proc. Natl. Acad. Sci. USA* 105, 5093-5098. 10.1073/pnas.0706197105 18362351 PMC2278217

[BIO058524C32] Scouten, C. W. (2010). *Frozen Section Technique in the Animal Research Setting. A Practical Guide to Frozen Section Technique*. New York: Springer.

[BIO058524C33] Sørensen, J. G. and Holmstrup, M. (2011). Cryoprotective dehydration is widespread in Arctic springtails. *J. Insect Physiol.* 57, 1147-1153. 10.1016/j.jinsphys.2011.03.001 21396373

[BIO058524C34] Storey, K. B. and Storey, J. M. (2012). Insect cold hardiness: metabolic, gene, and protein adaptation. *Can. J. Zool.* 90, 456-475. 10.1139/z2012-011

[BIO058524C35] Storey, K. B. and Storey, J. M. (2013). Molecular biology of freezing tolerance. *Compr. Physiol.* 3, 1283-1308. 10.1002/cphy.c130007 23897687

[BIO058524C36] Suzuki, D., Miyamoto, T., Kikawada, T., Watanabe, M. and Suzuki, T. (2014). A leech capable of surviving exposure to extremely low temperatures. *PLoS ONE* 9, e86807. 10.1371/journal.pone.0086807 24466250 PMC3899358

[BIO058524C37] Varki, A., Cummings, R., Esko, . J., Freeze, H. H., Stanley, P., Bertozzi, C. R., Hart, G. W. and Etzler, M. E. (2009). Chapter 16: Proteoglycans and Sulfated Glycosaminoglycans In *Essentials of Glycobiology Edition* ( J. D. Esko, K. Kimata and U. Lindahl). Cold Spring Harbor Laboratory Press, PMID: 20301236. 20301236

[BIO058524C38] Watanabe, M., Kikawada, T., Minagawa, N., Yukuhiro, F. and Okuda, T. (2002). Mechanism allowing an insect to survive complete dehydration and extreme temperatures. *J. Exp. Biol.* 205, 2799-2802. 10.1242/jeb.205.18.2799 12177145

[BIO058524C39] Wełnicz, W., Grohme, M. A., Kaczmarek, Ł., Schill, R. O. and Frohme, M. (2011). Anhydrobiosis in tardigrades—the last decade. *J. Insect Physiol.* 57, 577-583. 10.1016/j.jinsphys.2011.03.019 21440551

[BIO058524C40] Wharton, D. A. (2003). The environmental physiology of Antarctic terrestrial nematodes: a review. *J. Comp. Physiol. B.* 173, 621-628. 10.1007/s00360-003-0378-0 14615899

[BIO058524C41] Wright, J. C. (1989a). Desiccation tolerance and water-retentive mechanisms in Tardigrades. *J. Exp. Biol.* 142, 267-292. 10.1242/jeb.142.1.267

[BIO058524C42] Wright, J. C. (1989b). The tardigrade cuticle II. Evidence for a dehydration-dependent permeability barrier in the intracuticle. *Tissue Cell* 21, 263-279. 10.1016/0040-8166(89)90071-2 18620263

[BIO058524C43] Wright, J. C. (2001). Cryptobiosis 300 years on from van leuwenhoek: what have we learned about tardigrades? *Zool. Anz. A J. Comp. Zool.* 240, 563-582. 10.1078/0044-5231-00068

[BIO058524C44] Yamaguchi, A., Tanaka, S., Yamaguchi, S., Kuwahara, H., Takamura, C., Imajoh-Ohmi, S., Horikawa, D. D., Toyoda, A., Katayama, T., Arakawa, K. et al. (2012). Two novel heat-soluble protein families abundantly expressed in an anhydrobiotic tardigrade. *PLoS ONE* 7, e44209. 10.1371/journal.pone.0044209 22937162 PMC3429414

[BIO058524C45] Yang, T. (1996). *Fauna Sinica Annelida Hirudinea*, pp. 84-87. Beijing, PRC: Science Press. ISBN 7-03-004560-2/Q.565.

[BIO058524C46] Yin, H., Nie, L., Zhao, F., Zhou, H., Li, H., Dong, X., Zhang, H., Wang, Y., Shi, Q. and Li, J. (2016). De novo assembly and characterization of the Chinese three-keeled pond turtle (*Mauremys reevesii*) transcriptome: presence of longevity-related genes. *PeerJ* 4, e2062. 10.7717/peerj.2062 27257545 PMC4888314

[BIO058524C47] Zachariassen, K. E. (1985). Physiology of cold tolerance in insects. *Physiol. Rev.* 65, 799-832. 10.1152/physrev.1985.65.4.799 3903795

[BIO058524C48] Zhao, E. and Adler, K. (1993). *Herpetology of China*. Soc. Stud. Amph. Rept. Contr. Herpetol. 10.

